# Reduction of Peristalsis-Related Streak Artifacts on the Liver with Dual-Layer Spectral CT

**DOI:** 10.3390/diagnostics12040782

**Published:** 2022-03-23

**Authors:** Sergio Grosu, Zhen J. Wang, Markus M. Obmann, Mark D. Sugi, Yuxin Sun, Benjamin M. Yeh

**Affiliations:** 1 Department of Radiology and Biomedical Imaging, University of California, San Francisco, 513 Parnassus Ave., San Francisco, CA 94143, USA; zhen.wang@ucsf.edu (Z.J.W.); markus.obmann@usb.ch (M.M.O.); mark.sugi@ucsf.edu (M.D.S.); yuxin.sun@ucsf.edu (Y.S.); 2 Department of Radiology, University Hospital, LMU Munich, Marchioninistr 15, 81377 Munich, Germany; 3 Department of Radiology and Nuclear Imaging, University Hospital Basel, Petersgraben 4, 4051 Basel, Switzerland

**Keywords:** tomography, X-ray computed, DECT, humans, abdomen, liver, artifacts, peristalsis

## Abstract

Background: Peristalsis-related streak artifacts on the liver compromise image quality and diagnostic accuracy. Purpose: To assess dual-layer spectral-detector computed tomography (CT) image reconstructions for reducing intestinal peristalsis-related streak artifacts on the liver. Methods: We retrospectively evaluated 220 contrast-enhanced abdominal dual-energy CT scans in 131 consecutive patients (mean age: 68 ± 10 years, 120 men) who underwent routine clinical dual-layer spectral-detector CT imaging (120 kVp, 40 keV, 200 keV, virtual non-contrast (VNC), iodine images). Two independent readers evaluated bowel peristalsis streak artifacts on the liver qualitatively on a five-point Likert scale (1 = none to 5 = severe) and quantitatively by depth of streak artifact extension into the liver and measurements of Hounsfield Unit and iodine concentration differences from normal liver. Artifact severity between image reconstructions were compared by Wilcoxon signed-rank and paired t-tests. Results: 12 scans were excluded due to missing spectral data, artifacts on the liver originating from metallic foreign materials, or oral contrast material. Streak artifacts on the liver were seen in 51/208 (25%) scans and involved the left lobe only in 49/51 (96%), the right lobe only in 0/51 (0%), and both lobes in 2/51 (4%) scans. Artifact frequency was lower in iodine than in 120 kVp images (scans 18/208 vs. 51/208, *p* < 0.001). Artifact severity was less in iodine than in 120 kVp images (median score 1 vs. 3, *p* < 0.001). Streak artifact extension into the liver was shorter in iodine than 120 kVp images (mean length 2 ± 4 vs. 12 ± 5 mm, *p* < 0.001). Hounsfield Unit and iodine concentration differed significantly between bright streak artifacts and normal liver in 120 kVp, 40 keV, 200 keV, and VNC images (*p* < 0.001, each), but not in iodine images (*p* = 0.23). Conclusion: Intestinal peristalsis-related streak artifacts commonly affect the left liver lobe at CT and can be substantially reduced by viewing iodine dual-energy CT image reconstructions.

## 1. Introduction

Cancer is the second leading cause of death among men and women in the United States [1]. In oncologic cases, metastases and primary tumors commonly affect the liver. Computed tomography (CT) is often the imaging modality of choice for liver tumor detection, characterization, treatment planning, and response monitoring.

Since liver lesions may be small or have poor contrast resolution, optimization of image quality is critical. Artifacts in CT may degrade image quality and compromise diagnostic accuracy, potentially leading to repeated scans and inappropriate patient management [2,3]. Several causes for CT artifacts are known and can be classified into physics-based, scanner-based, reconstruction-based, and patient-based artifacts [2]. Patient-based artifacts are related to metallic implants or motion of the patient [2,4]. Especially motion artifacts related to cardiac pulsation or intestinal peristalsis are a challenge in CT because they cannot be reduced by patient instruction, patient positioning, or sedation.

Intestinal peristalsis-related artifacts are predominantly due to the movement of intraluminal gas in the stomach or bowel during CT scanning, and can appear as bright or dark streaks in CT images [5,6,7]. To reduce this phenomenon, different methods have been developed. Gastric and intestinal motility can be reduced through fasting prior to the examination and the administration of anti-peristaltic drugs such as anticholinergics [8,9]. Additionally, rapid scanning and software-based correction algorithms can be used to reduce the frequency and severity of peristalsis-related artifacts [2,3,10,11]. Nonetheless, peristalsis motion artifacts remain common in CT scans of the abdomen with a prevalence of about 30% to 70% [3,6]. The liver is often affected by peristalsis-related artifacts due to its anatomical proximity to the stomach [6].

Dual-energy computed tomography (DECT) uses high and low X-ray photon energy spectra to differentiate materials that exhibit varying attenuation properties at different photon energies [12,13,14]. Multiple studies have shown the ability of DECT to reduce CT artifacts such as from metal [15,16,17,18,19]. However, data on peristalsis-related artifact reduction are scarce. Initial studies indicate that these artifacts can be reduced by viewing certain DECT image reconstructions, but there is a lack of studies focusing on the liver, the non-bowel organ most frequently affected by peristalsis-related artifacts. [6,20].

The aim of our study was to assess the performance of dual-layer spectral-detector CT scanner DECT image reconstructions for the reduction of peristalsis-related streak artifacts on the liver.

## 2. Materials and Methods

Our study was compliant with the Health Insurance Portability and Accountability Act. Our institutional review board waived the need for informed consent because our study analyzed retrospective anonymized data acquired for routine clinical care. Funding for this study was received from Philips Healthcare, Cleveland, OH, USA and the National Institutes of Health as part of research grants. The funders had no role in the design of the study; in the collection, analyses, or interpretation of data; in the writing of the manuscript, and in the decision to publish the results.

### 2.1. Study Population

In our retrospective analysis, we evaluated all routine clinical contrast-enhanced CT scan of the abdomen performed on a single dual-layer spectral-detector CT scanner during the time period from 13 September 2017 to 1 April 2018. Fasting prior to CT scanning was not required. No antispasmodic medication was given. CT scans of patients <18 years-of-age or with artifacts in the liver originating from abdominal or thoracic metallic foreign materials or oral contrast material in the stomach or bowel were excluded. CT scans with missing spectral data were excluded. For each CT scan, the scan date, indication, patient age, and patient sex were recorded.

### 2.2. CT Image Acquisition

All CT image data sets were acquired on a dual-layer spectral-detector DECT scanner (IQon; Philips Healthcare, Cleveland, OH, USA) in helical scanning mode, axial acquisition plane, collimation of 0.625 mm, tube voltage of 120 kVp, tube current–time product reference values of 70 mAs using automatic tube current adaption, and a mean CTDIvol of 13.0 mGy. Spectral DECT images and true conventional 120 kVp CT images were acquired simultaneously. Images were reconstructed at a slice thickness of 2.5 mm and 2.5 mm reconstruction increment using a standard soft-tissue kernel as conventional 120 kVp images, 40 keV monoenergetic images, 200 keV monoenergetic images, virtual non-contrast (VNC) images, and iodine density images.

### 2.3. Image Analysis

Image analysis was performed on a standard picture archiving and communication system workstation using a commercially available dedicated post-processing software (IntelliSpace Portal 11.1; Philips Medical Systems Nederland B.V., Best, The Netherlands) by two independent radiologists (Reader A: S.G., 4 years of experience in abdominal radiology) (Reader B: B.M.Y., 18 years of experience in abdominal radiology, respectively). All CT scans were viewed in axial reconstructions of 2.5 mm slice thickness. All conventional 120 kVp images were viewed in a standard abdominal window width of 400 HU and window level of 40 HU for the presence or absence of peristalsis-related streak artifacts on the liver. When a peristalsis-related streak artifact was seen, the organ from which the peristalsis-related streak artifacts originated was recorded. In order to assess inter-reader and intra-reader variability, all CT scans with streak artifacts were evaluated independently by Reader A and B, and the first 50% of all consecutive CT scans were evaluated again four weeks after the first reading by Reader A.

### 2.4. Qualitative Evaluation of Streak Artifacts in the Liver

Peristalsis-related streak artifact severity on the liver on conventional 120 kVp images, 40 keV and 200 keV monoenergetic image reconstructions, VNC, and iodine image reconstructions was qualitatively evaluated on a five-point Likert scale: 1 = Absence of streak artifact on the liver; 2 = Visible streak artifact with no effect on diagnosis on the liver; 3 = Moderate streak artifact that may decrease confidence in diagnosing a 0.5 to 0.9 cm liver lesion; 4 = Distinct streak artifact that prevents the diagnosis of a 0.5 to 0.9 cm liver lesion and that may decrease confidence in diagnosing a ≥1.0 cm liver lesion; 5 = Severe streak artifact that prevents the diagnosis of a ≥1.0 cm liver lesion. If one or both readers scored an artifact as visible, it was considered as present. The qualitative artifact scoring system was created by all readers in consensus and example non-study cases were discussed together before the actual reading.

### 2.5. Quantitative Evaluation of Streak Artifacts in the Liver

To quantitatively evaluate the severity of the peristalsis-related streak artifact on the liver, the depth of streak artifact extension into the liver parenchyma from the liver capsule was measured in millimeters on axial conventional 120 kVp images, 40 keV and 200 keV monoenergetic image reconstructions, VNC, and iodine image reconstructions.

In addition, circular regions of interest (ROI) were placed manually in the most visibly bright area of the streak artifact, in the most visibly dark area of the streak artifact, and in the neighboring liver parenchyma not affected by artifacts in axial conventional 120 kVp images. Areas of inhomogeneity due to vessels, tissue borders, or partial volume effect were avoided. The ROIs were automatically populated simultaneously onto the axial 40 keV and 200 keV monoenergetic image reconstructions, VNC, and iodine image reconstructions with identical ROI sizes in identical anatomical ROI locations. Quantitative ROI measurements were given in HU (120 kVp, 40 keV, 200 keV, and VNC image reconstructions) or iodine concentrations in mg/mL (iodine image reconstructions). In each image dataset, comparisons of quantitative ROI measurements were performed between the artifact measurements and the neighboring liver parenchyma not affected by artifacts which served as reference tissue.

### 2.6. Statistical Analysis

Categorical variables were expressed as frequencies and percentages, and continuous variables as mean ± standard deviation. Testing for normality was performed using the Shapiro-Wilk test.

Inter-reader and intra-reader agreement for qualitative and quantitative streak artifact evaluation was calculated using weighted Cohen’s kappa-coefficients and intra-class correlation coefficients (ICC), respectively [21,22,23].

The Wilcoxon signed rank test was performed to compare qualitative streak artifact scores and the depth of streak artifact extension into the liver between conventional 120 kVp images and 40 keV monoenergetic image reconstructions, 200 keV monoenergetic image reconstructions, VNC image reconstructions, and iodine image reconstructions.

The paired t-test was used to assess the relationship between quantitative values (HU, iodine concentration) of peristalsis-related streak artifacts and the neighboring liver parenchyma not affected by artifacts in conventional 120 kVp images, 40 keV and 200 keV monoenergetic image reconstructions, VNC, and iodine image reconstructions.

*p*-values < 0.05 were considered to denote statistical significance. Statistical analysis was performed with the open-source software RStudio Version 1.4.1103 (RStudio Team (2020), RStudio: Integrated Development for R. RStudio, PBC, Boston, MA, USA).

## 3. Results

### 3.1. Study Population

**Figure 1:**
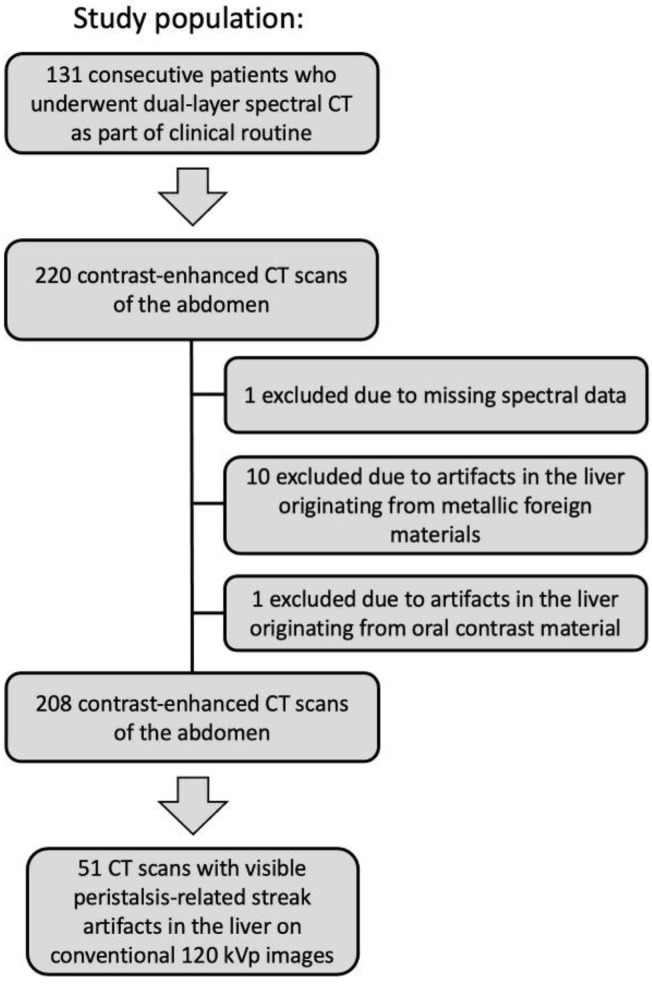
Flow diagram of the study population. CT (Computed tomography).

We evaluated 220 contrast-enhanced (42/220 (19%) arterial phase, 119/220 (54%) venous phase, 59/220 (27%) delayed phase) CT scans of the abdomen in 131 consecutive patients (mean age: 68 ± 10 years, 120 men). The indications for CT scanning were 157/220 (71%) “possible or known malignancy”, 31/220 (14%) “abdominal pain”, 12/220 (6%) “infection”, and 20/220 (9%) “other”. 1/220 (1%) CT scan was excluded due to missing spectral data, 10/220 (5%) CT scans were excluded due to artifacts on the liver originating from metallic foreign materials, 1/220 (1%) CT scan was excluded due to artifacts on the liver originating from oral contrast material, resulting in 208 CT scans in 122 patients (see Figure 1). Intestinal peristalsis-related streak artifacts on the liver were present in 51/208 (25%) CT scans of 40/122 (33%) patients, and involved the left liver lobe only in 49/51 (96%), the right liver lobe only in 0/51 (0%), and both liver lobes in 2/51 (4%) CT scans. The origin of peristalsis artifact on the liver was the stomach in 46/51 (90%) and the transverse colon in 5/51 (10%) CT scans. 5/51 (10%) CT scans with peristalsis-related streak artifacts on the liver were acquired in the arterial phase, 30/51 (59%) in the venous phase, and 16/51(31%) in the delayed phase.

**Figure 2:**
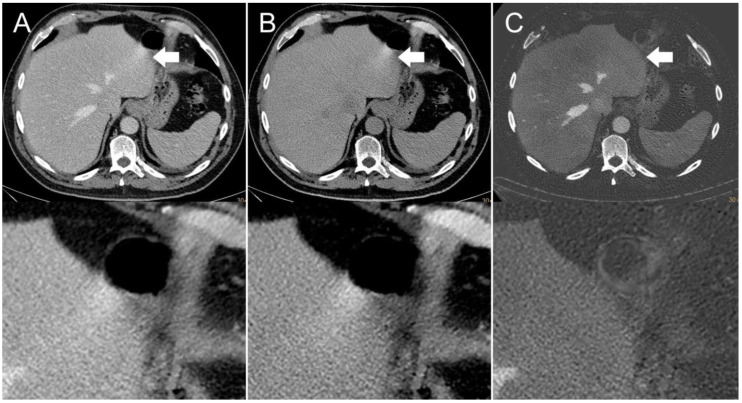
Axial contrast-enhanced CT scan of a 65-year-old man. Streak artifact (indicated by arrows) on the left liver lobe originating from peristaltic motion of the stomach visible in the (**A**) conventional 120 kVp image and (**B**) virtual non-contrast (VNC) image reconstruction. The streak artifact is markedly reduced in the (**C**) iodine image reconstruction, resulting in a better visibility of the underlying liver tissue.

**Figure 3:**
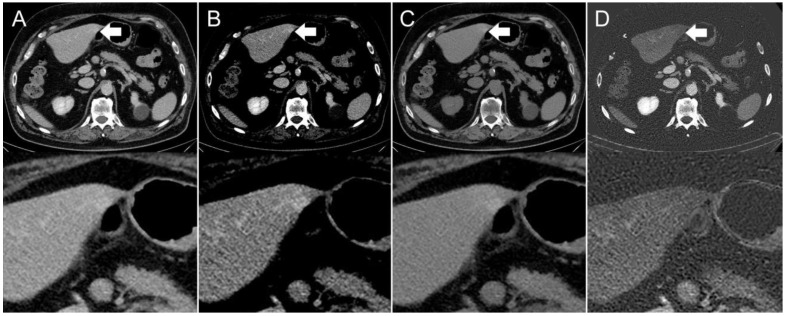
Axial contrast-enhanced CT scan of a 67-year-old man. Hyperdensity (indicated by arrows) on the left liver lobe in the (**A**,**B**) conventional 120 kVp image using an (**A**) abdomen window and (**B**) liver window, which might be interpreted as a hyperenhancing liver lesion. The (**C**) virtual non-contrast (VNC) image reconstruction and (**D**) iodine image reconstruction reveal that it is not a hyperenhancing liver lesion, but a streak artifact originating from the stomach antrum.

**Figure 4:**
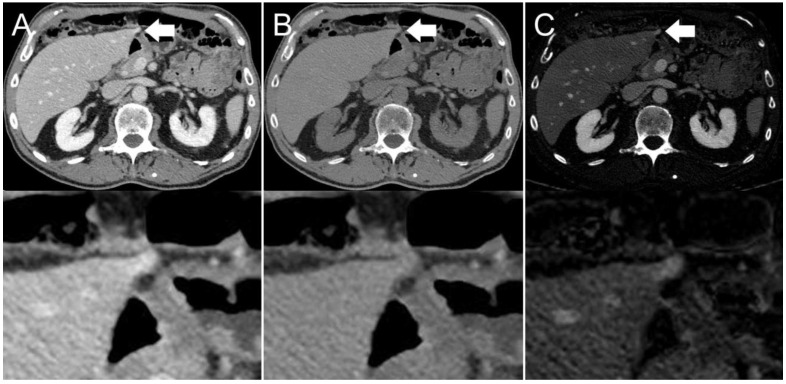
Axial contrast-enhanced CT scan of an 86-year-old man. Hyperdensity (indicated by arrows) on the left liver lobe in the (**A**) conventional 120 kVp image, which might be interpreted as a peristalsis-related streak artifact originating from the stomach. The (**B**) virtual non-contrast (VNC) image reconstruction and (**C**) iodine image reconstruction reveal that it is not a streak artifact, but a hyperenhancing liver lesion.

The presence of visible intestinal peristalsis-related streak artifacts on the liver was significantly lower (*p* < 0.001) in the iodine image reconstructions with 18/208 (9%) compared with the conventional 120 kVp images with 51/208 (25%) (see Figure 2, Figure 3 and Figure 4). The presence of visible peristalsis-related streak artifacts on the liver was not significantly lower in 40 keV monoenergetic image reconstructions with 48/208 (23%) (*p* = 0.15), 200 keV monoenergetic image reconstructions with 51/208 (25%) (*p* = 1.0), or VNC image reconstructions with 50/208 (24%) (*p* = 1.00) compared with conventional 120 kVp images.

### 3.2. Qualitative Evaluation of Peristalsis-Related Streak Artifacts in the Liver

The inter-reader agreement in the five-point Likert scale rating of peristalsis-related streak artifact severity in the liver was moderate (kappa-coefficient of 0.46, *p* < 0.001), and the intra-reader agreement was almost perfect (kappa-coefficient of 0.89 *p* < 0.001).

For Reader A and B, qualitative artifact scores were significantly lower (*p* < 0.001, each) for iodine image reconstructions (Reader A, median score: 1, range: 1–3) (Reader B, median score: 1, range: 1–4) compared with conventional 120 kVp images (Reader A, median score: 3, range: 2–5) (Reader B, median score: 3, range: 2–5).

No significant differences were found in qualitative artifact scores of Reader A between 40 keV monoenergetic image reconstructions (median score: 3, range: 1–5) and conventional 120 kVp images (median score: 3, range: 2–5) (*p* = 0.67), 200 keV monoenergetic image reconstructions (median score: 3, range: 2–5) and conventional 120 kVp images (*p* = 0.77), VNC image reconstructions (median score: 3, range: 1–5), and conventional 120 kVp images (*p* = 0.78).

For Reader B, qualitative artifact scores were significantly lower (*p* < 0.001) for 40 keV monoenergetic image reconstructions (median score: 3, range: 1–5) compared with conventional 120 kVp images (median score: 3, range: 2–5), and significantly higher (*p* < 0.001, *p* = 0.003, respectively) for 200 keV monoenergetic image reconstructions (median score: 4, range: 1–5) and VNC image reconstructions (median score: 4, range: 1–5) compared with conventional 120 kVp images. Artifact scores are presented in Table 1.

### 3.3. Quantitative Evaluation of Peristalsis-Related Streak Artifacts in the Liver

The inter-reader agreement in quantitative assessment of peristalsis-related streak artifacts in the liver was excellent (ICC of 0.97 *p* < 0.001), and the intra-reader agreement was excellent (ICC of 0.98, *p* < 0.001).

**Figure 5:**
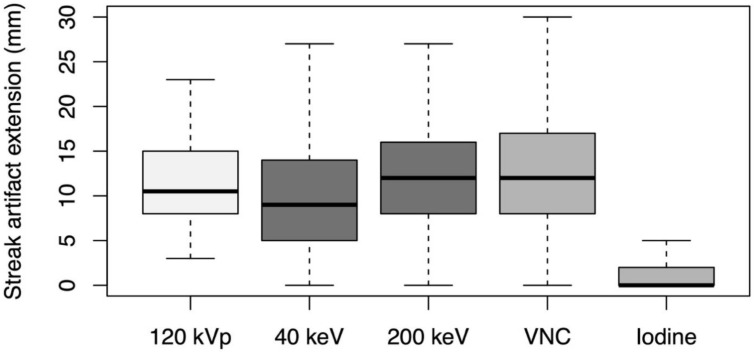
Depth of extension of peristalsis-related streak artifacts into the liver parenchyma measured on axial conventional 120 kVp images, 40 keV monoenergetic images, 200 keV monoenergetic images, virtual non-contrast (VNC) images, and iodine images.

The depth of extension of peristalsis-related streak artifact into the liver was significantly shorter (*p* < 0.001, each) for iodine image reconstructions (mean length: 2 ± 4 mm) and 40 keV monoenergetic image reconstructions (mean length: 10 ± 7 mm) compared with conventional 120 kVp images (mean length: 12 ± 5 mm) (see Figure 5). The depth of extension of peristalsis-related streak artifact into the liver was significantly longer (*p* < 0.001, each) for 200 keV monoenergetic image reconstructions (mean length: 13 ± 7 mm) and VNC image reconstructions (mean length: 13 ± 7 mm) compared with conventional 120 kVp images.

In iodine image reconstructions the mean ROI measurements of iodine concentrations were not significantly different in the bright streak artifact components compared to the neighboring liver parenchyma not affected by artifacts (*p* = 0.23), as opposed to the dark artifact components (*p* < 0.001). The mean ROI measurements of HU were significantly different in the bright and dark streak artifact components compared with the neighboring liver parenchyma not affected by artifacts in conventional 120 kVp images (*p* < 0.001, each), 40 keV monoenergetic image reconstructions (*p* < 0.001, each), 200 keV monoenergetic image reconstructions (*p* < 0.001, each), and VNC image reconstructions (*p* < 0.001, each). ROI measurements are presented in Table 2.

## 4. Discussion

We found that peristalsis-related streak artifacts on the liver at dual-layer spectral-detector CT were significantly less frequent and less severe when the liver was viewed using iodine image reconstructions than on conventional 120 kVp images (*p* < 0.001 for each). The depth of streak artifact extension into the liver was significantly less (*p* < 0.001) in iodine image reconstructions than in conventional 120 kVp images. Additionally, ROI measurements of iodine concentrations were not significantly different (*p* = 0.23) between bright streak artifacts and the neighboring liver parenchyma not affected by artifacts, as opposed to conventional 120 kVp (*p* < 0.001) images. However, ROI measurements of iodine concentrations were significantly different (*p* < 0.001) between dark streak artifact components and the neighboring liver parenchyma not affected by artifacts, indicating that in iodine image reconstructions the attenuation of the less prominent dark streak artifact components is not as effective as that of the bright components.

Our findings support the notion that peristalsis-related streak artifacts have the characteristics of a highly attenuating tissue with spectral properties similar to water, as these artifacts affect the 40 keV and 200 keV monoenergetic image reconstructions to a similar degree and appear intensely on the VNC image but not the iodine image reconstructions [6]. Since iodine image reconstructions depict voxels with a relatively large decrease in HU between low kVp and high kVp images, streak artifacts are largely absent in the iodine images, revealing the underlying liver tissue enhancement [12]. Our study shows that iodine image reconstructions may improve diagnostic accuracy for cases with streak artifacts on the liver. Parts of the liver that are not clearly visible on conventional 120 kVp images because of peristalsis-related artifacts can be reliably evaluated by viewing iodine images of the same CT scan.

Our results are in line with prior studies assessing the potential of motion artifact reduction in DECT [6,20,24]. Winklhofer et al. showed in a peristalsis phantom and in 100 patients examined on a rapid-kV switching CT scanner that peristalsis-related streak artifacts seen in 70 keV, 120 keV, and VNC image reconstructions were substantially reduced in iodine image reconstructions (*p* < 0.001, respectively) [6]. That study focused on general bowel peristalsis artifact incidence, with only a passing mention of the frequency of peristalsis artifacts on the liver [6]. Our study adds to the field by providing a more detailed evaluation of the depth of extension and severity of peristalsis-related artifacts on the liver at clinical DECT, including a five-point Likert scale based on oncologic liver evaluation, and assessment of a different DECT scanner platform, a dual-layer spectral-detector CT scanner. The rapid kV-switching DECT scanner used an X-ray tube that switched between a high- and low-energy spectrum, whereas the dual-layer spectral-detector DECT scanner performed spectral separation of a conventional polychromatic X-ray beam at the detector level [13,20,25]. In addition to studying iodine and VNC image reconstructions, we also assessed conventional 120 kVp images instead of monoenergetic 70 keV 120 kVp-like images, as well as 40 and 200 keV monoenergeic images, allowing for a more thorough comparison of DECT image reconstructions with conventional CT images.

Our study has limitations. An effective blinding of the readers to the different image reconstructions was not possible, as the appearances of the monoenergetic images and material decomposition images are very characteristic. Our study only included scans from a dual-layer spectral-detector CT, limiting the applicability of our results to other DECT scanner models such as dual-source CT scanners, rapid-kV switching CT scanners, or split filter CT scanners. However, the use of a single CT scanner eliminated inter-scanner variability bias from our analysis. Another limitation is that the proportion of men in our study population was disproportionately high compared with the US population but reflects the population of our military hospital. Future studies with broader populations and with comparisons to other DECT scanner platforms are warranted.

In conclusion, peristalsis-related streak artifacts on the liver in conventional CT images were substantially reduced in iodine image reconstructions acquired on a dual-layer spectral-detector DECT, resulting in improved reader confidence.

## Figures and Tables

**Table 1:** Qualitative score of peristalsis-related streak artifact severity on the liver of Reader A and B in conventional 120 kVp images, 40 keV monoenergetic images, 200 keV monoenergetic images, virtual non-contrast (VNC) images, and iodine images.

Reader A					
Image Reconstructions	Score				
	1	2	3	4	5
120 kVp	0/51 (0%)	22/51 (43%)	18/51 (35%)	9/51 (18%)	2/51 (4%)
40 keV	3/51 (6%)	19/51 (37%)	17/51 (33%)	5/51 (10%)	7/51 (14%)
200 keV	0/51 (0%)	23/51 (45%)	15/51 (29%)	11/51 (22%)	2/51 (4%)
VNC	1/51 (2%)	22/51 (43%)	14/51 (27%)	12/51 (24%)	2/51 (4%)
iodine	33/51 (65%)	14/51 (27%)	4/51 (8%)	0/51 (0%)	0/51 (0%)
**Reader B**					
**Image Reconstructions**	**Score**				
	**1**	**2**	**3**	**4**	**5**
120 kVp	0/51 (0%)	9/51 (18%)	19/51 (37%)	11/51 (22%)	12/51 (24%)
40 keV	10/51 (20%)	11/51 (22%)	16/51 (31%)	4/51 (8%)	10/51 (20%)
200 keV	1/51 (2%)	4/51 (8%)	16/51 (31%)	12/51 (24%)	18/51 (35%)
VNC	1/51 (2%)	5/51 (10%)	16/51 (31%)	12/51 (24%)	17/51 (33%)
iodine	42/51 (82%)	4/51 (8%)	2/51 (4%)	3/51 (6%)	0/51 (0%)

Score 1 = Absence of streak artifact on the liver; Score 2 = Visible streak artifact with no effect on diagnosis on the liver; Score 3 = Moderate streak artifact that may decrease confidence in diagnosing a 0.5 to 0.9 cm liver lesion; Score 4 = Distinct streak artifact that prevents the diagnosis of a 0.5 to 0.9 cm liver lesion and may decrease confidence in diagnosing a ≥1.0 cm liver lesion; Score 5 = Severe streak artifact that prevents the diagnosis of a ≥1.0 cm liver lesion.

**Table 2:** Quantitative measurements of peristalsis-related streak artifacts on the liver on conventional 120 kVp images, 40 keV monoenergetic images, 200 keV monoenergetic images, virtual non-contrast (VNC) images, and iodine images.

Image Reconstructions	Quantitative Artifact Measurements			*p*-Values	
	Mean ROI_max_	Mean ROI_min_	Mean ROI_ref_	Mean ROI_max_ Compared with Mean ROI_ref_	Mean ROI_min_ Compared with Mean ROI_ref_
120 kVp	129 HU	80 HU	101 HU	*p* < 0.001	*p* < 0.001
40 keV	232 HU	168 HU	201 HU	*p* < 0.001	*p* < 0.001
200 keV	91 HU	48 HU	67 HU	*p* < 0.001	*p* < 0.001
VNC	87 HU	45 HU	61 HU	*p* < 0.001	*p* < 0.001
iodine	1.72 mg/mL	1.44 mg/mL	1.68 mg/mL	*p* = 0.23	*p* < 0.001

ROI_max_ (regions of interest measurement in the most visibly bright area of the streak artifact). ROI_min_ (regions of interest measurement in the most visibly dark area of the streak artifact). ROI_ref_ (regions of interest measurement in the neighboring liver parenchyma not affected by artifacts).

## Data Availability

Data are available upon reasonable request. Requests should be sent to the corresponding author and are subject to approval.

## Abbreviations

CT (Computed tomography), CTDIvol (Computed tomography dose index), DECT (Dual-energy computed tomography), HU (Hounsfield Units), keV (Kiloelectronvolt), kVp (Peak kilovoltage).
